# A novel modified penile disassembly procedure for isolated male epispadias repair: preliminary outcomes

**DOI:** 10.3389/fped.2024.1371576

**Published:** 2024-08-22

**Authors:** Fuming Deng, Wen Fu, Guochang Liu, Liangzhong Sun

**Affiliations:** ^1^ Pediatrics Department, Nanfang Hospital, Southern Medical University, Guangzhou, Guangdong, China; ^2^ Urology Department, Guangzhou Women and Children’s Medical Center, Guangzhou, Guangdong, China

**Keywords:** epispadias, urethral diseases, urethral reconstruction, male urogenital diseases, prognosis

## Abstract

**Purpose:**

This study aimed to evaluate the preliminary outcome of a novel modification of a penile disassembly procedure.

**Methods:**

We retrospectively reviewed the data of 15 patients with isolated male epispadias (IME) who underwent a modified penile disassembly procedure. This modification involved leaving the glans connected while dissecting the urethral plate from the corpus cavernosum. This approach reserves the bundles of the anastomosis at the glans, which can prevent ischemic changes.

**Results:**

One of the first two patients experienced glans ischemic changes on the first day after the operation, which ultimately resulted in the loss of half of the hemiglans. Urethral plate shortening was observed in two patients and was addressed with a transverse preputial island flap. Two patients developed a penopubic urethral fistula, which was repaired 6 months postoperatively. Of the 15 patients, 10 (66%) were continent or social continent, while 4 were incontinent and needed bladder neck reconstruction. One patient had not yet reached the age for continence evaluation. Additionally, Two patients had a residual dorsal curvature of approximately 10–15° and were advised to undergo continuous follow-up.

**Conclusions:**

The modified penile disassembly procedure is a simple, effective, and safe method for IME repair with an acceptable penile appearance and complication rate.

## Introduction

Isolated male epispadias (IME) is a rare congenital urogenital malformation with the absence of bladder exstrophy. Its repair is still a challenge for pediatric urological surgeons (1). The repair of the bladder exstrophy–epispadias complex (BEEC) has progressed greatly since Cantwell (2) and Mitchell and Bagli (3) presented methods based on penile disassembly. However, the outcome of IME has rarely been reported. Moreover, the non-exposed bladder makes IME different from BEEC in terms of urinary control, volitional voiding, and cosmetic results, while having a more satisfactory outcome in terms of bladder capacity (4, 5). Despite the versatility of the complete penile disassembly procedure (3, 6), ischemic changes at the hemiglans and in the penile skin, with an incidence ranging from 20% to 50% (7), and sometimes complete loss of the hemiglans have been reported in the literature (8). Moreover, urethral shortening seems to be an inherent complication that can be found in 30%–70% of patients who undergo a complete penile disassembly procedure. To avoid the disadvantages of the complete penile disassembly procedure and simplify the surgical technique, we introduced a modification that keeps the glans connected. This study aimed to report our preliminary experience with this modification for IME repair.

## Materials and methods

We retrospectively collected data from a consecutive series of 15 patients with IME between January 2016 and December 2019. All patients underwent the modified penile disassembly procedure. According to the meatus location, the patients were classified as having penopubic epispadias (PPE), penile epispadias (PE), or glandular epispadias (GE). Of these patients, five had PPE, nine had PE, and one presented with GE.

All patients underwent genitourinary ultrasonography and voiding cystourethrography (VCUG) to evaluate the bladder capacity and reflux preoperatively and 6 months postoperatively.

Pediatric Penile Perception Score (PPPS) (9) was used to evaluate the postoperative appearance of the penis. Patients or their parents answered the PPPS questionnaires by mail or telephone.

Patients were categorized as continent, social continent, and incontinent. Continent patients had daytime dry intervals of >3 h and were dry during nighttime. Social continent patients had daytime dry intervals of >3 h and were wet during nighttime. Incontinent patients had wet days and nights with dry intervals of <3 h (10).

The median age at surgery was 30 months (range, 12–80 months). All patients underwent surgery via the same technique and stayed in the hospital for 5–6 days postoperatively. The penis dressing was removed on Days 4–5 after surgery, and catheter removal was carried out 11–14 days postoperatively in the outpatient clinic.

The paired *t*-test was employed to assess changes in bladder capacity across follow-up intervals. A *p*-value of <0.05 was deemed statistically significant for the analysis.

This research was approved by the institutional research ethics committee of the Guangzhou Women and Children's Medical Centers (Approval No. 31401). The need to obtain informed consent was waived because the study was retrospective.

## Surgical technique

**Figure 1:**
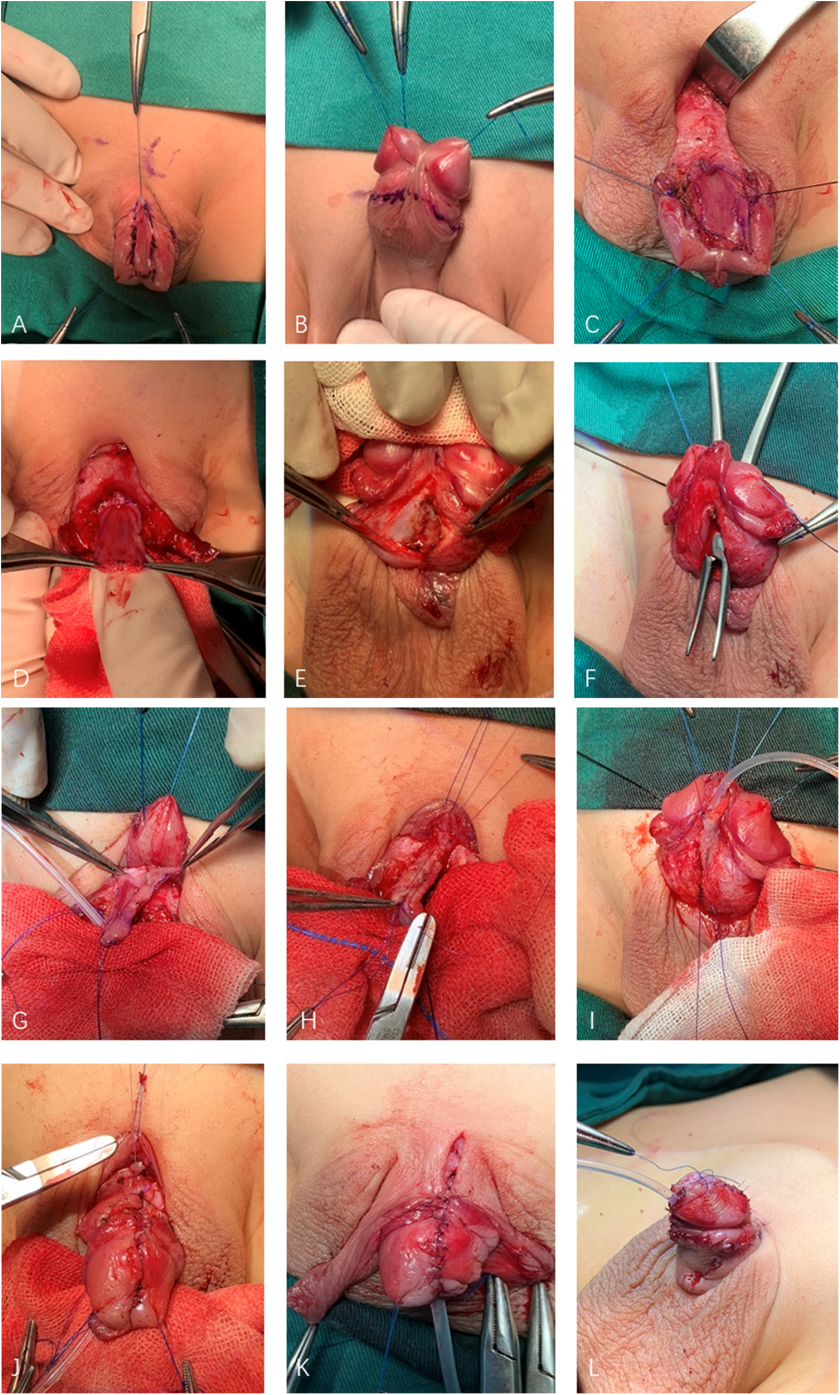
The surgical procedure of modified penile disassembly. **(A**,**B)** A U-shaped incision was made 2 mm proximal to the epispadiac meatus. **(C)** Deglove the penile skin. **(D)** The urethral plate was separated from the penile corporal. **(E**,**F)** The corpus cavernosum was separated proximally and distally the corpus cavernosum with the hemiglans remained connected. **(G**,**H)** The urethral plate transposed under the corpora cavernosa and tubularized. **(I)** The neourethra was fixed to the corpus cavernosum. **(J)** The corpora were internally rotated. **(K)** Penis was covered by reversed modified Byar's flaps. **(L)** Postoperative appearance.

Each patient was anesthetized by combined general anesthesia and a sacral block. Two traction 5-0 Prolene sutures were placed through the tip of the glans. A U-shaped incision was made 2 mm proximal to the epispadiac meatus (Figures 1A,B). An incision must be made middorsal to each corpus cavernosum to prevent injury to the neurovascular bundle. After degloving the penile skin (Figure 1C), the urethral plate was separated from the penile corporal by fine scissors, and we dissected the Buck's fascia as thick as possible to ensure a proper blood supply (Figure 1D). The boundary of the urethral plate dissection and tubularization for GE and PE patients was extended up to the verumontanum, whereas for PPE patients, it was limited to the proximal urethral opening. The dissection of the corpora initiates from the ventral side, carefully following the surface of Buck's fascia that envelops the corporeal bodies. This dissection continues along this plane until it emerges on the dorsal aspect of the penis, sandwiched between the corpora, starting with one side before moving to the other. Each corpus cavernosum thus was separated proximally and distally from the corpus cavernosum with the hemiglans remaining connected (Figures 1E,F). The urethral plate was transposed under the corpora cavernosa through the separated corpora and tubularized around a 6 F or 8 F catheter by interrupted sutures with 6-0 PDS to create the neourethra (Figures 1G–I). Attention must be paid to the neurovascular bundles, which are situated between Buck's fascia and the lateral corporeal wall, particularly as this arrangement differs from that of a typical penis. When the neurovascular bundles interfere with the internal rotation of the corpora, they should be meticulously dissected. This approach ensures that the neurovascular bundles remain intact and are not compromised by the incisions made in the corpora during the rotation process. The corpora were internally rotated and reapproximated with interrupted 4-0 polyglycate sutures on the dorsal surface to correct the curvature of the dorsal penis (Figure 1J). Ventral plication can be used to correct penile curvature when a dorsal curve cannot be corrected by corpora rotation. The neourethra was connected with interrupted sutures by 6-0 monoglycate ventrally to the corpora and brought to the glans to create a glanular meatus. Glanuloplasty was then performed by reshaping the glans tissue to obtain a conical appearance. The penis was covered by reversed modified Byar's flaps (Figure 1K) and had a good cosmetic appearance (Figure 1L). Additionally, a preputial island flap technique was employed to repair the distal urethral defect, as described by the previous study (11, 12). An island flap from the ventral prepuce was selected and measured to cover the gap of the urethral defect. Careful dissection was performed to free the preputial flap, preserving its blood supply, as per Duckett's method. The flap was rotated 180° and tubularied using 6-0 monoglycate. This tubularized graft was then utilized to repair the urethral defect.

## Results

The patients were routinely followed up 1, 3, and 6 months after surgery and later every year. The median follow-up was 22.5 months (range, 12–40 months).

All children had normal volitional voiding per urethra. The VCUG results showed that pubic diastasis was present in six (40%) patients, including five PPE patients and one PE patient. Unilateral vesicoureteral reflux (Grade I–II) was found in three PPE patients (20%). These patients with vesicoureteral reflux were monitored without intervention, as their urinary analysis was negative and no urinary infections were detected.

One of the first two patients experienced glans ischemic changes on the first day after the operation, which ultimately resulted in the loss of half of the hemiglans. Urethral plate shortening was found in two (13%) patients, which was addressed with a transverse preputial island flap (11, 12). Three patients had a penopubic urethral fistula that was repaired 6 months postoperatively. Two patients had a residual dorsal curvature of approximately 10–15° and were advised to undergo continuous follow-up. There was no urethral stenosis or glans dehiscence (Table 1).

**Table 1:** Complication of modified penile disassembly in IME repair.

Complication	No. of patients (*n* = 15)
Urethral fistula	3 (20%)
Glans ischemic change	1 (6.7%)
Residual dorsal curvature	2 (13%)
Urethral shortening	2 (13%)
Glans dehiscent	0
Urethral stricture	0
Totally complication	8 (53%)

**Figure 2:**
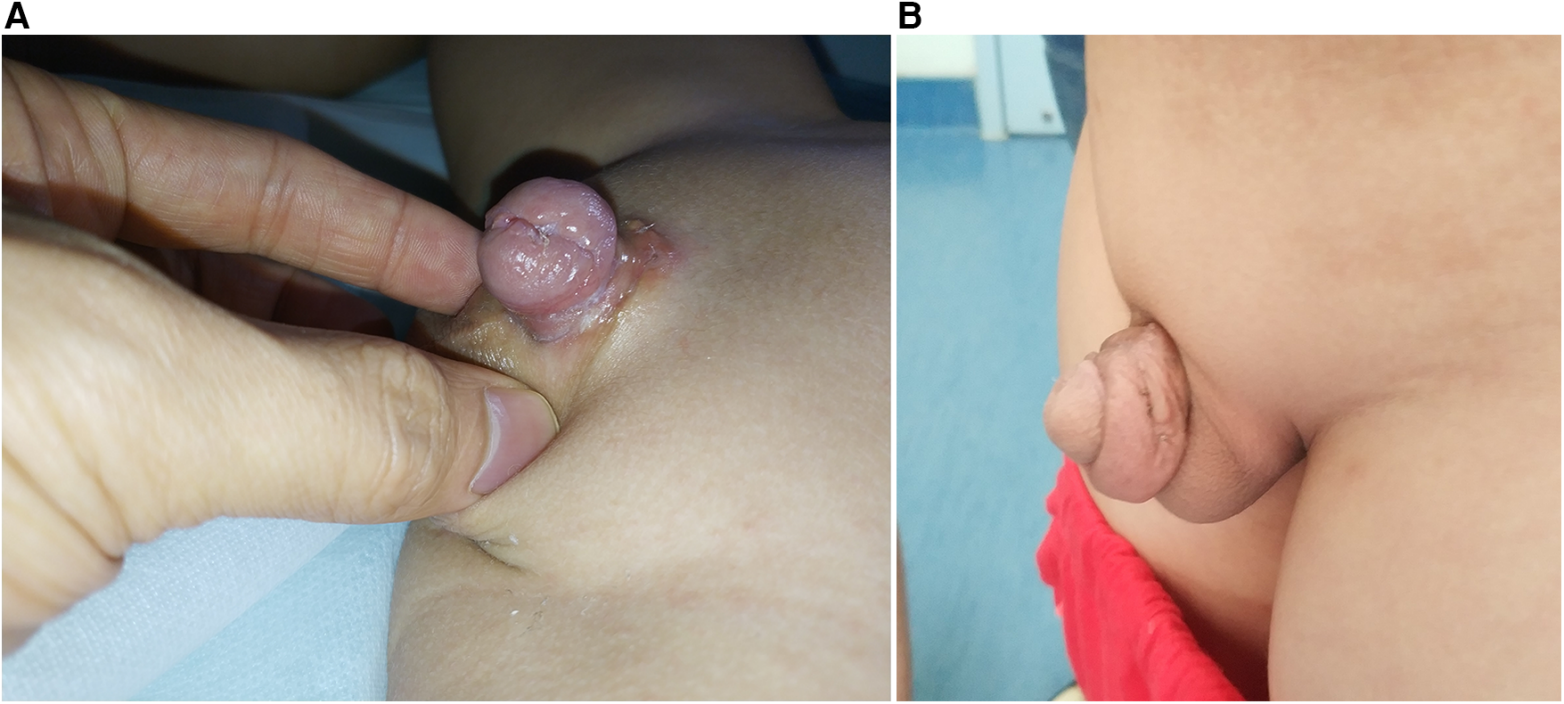
**(A**,**B)** Postoperative appearance 1 year after the modified penile disassembly procedure.

The overall PPPS was 8.80 ± 2.16. The PPPS result showed that the postoperative appearance of post-IME repair was acceptable (Table 2 and Figure 2).

**Table 2:** PPPS of IME patients.

	Mean	SD
Meatus	2.13	0.38
Glans	2.40	0.24
Shaft skin	2.06	0.46
General appearance	2.20	0.16
Overall PPPS	8.80	2.16

PPPS, pediatric penile perception score.

Of our 15 patients, 10 were continent or social continent, and 4 patients were incontinent and needed bladder neck reconstruction. The remaining patients had not yet reached the age at which continence can be evaluated. Both the GE and PE patients were continent or social continent, while four PPE patients required bladder neck reconstruction. One PPE patient has not yet reached the age needed to evaluate continence. The urinary function outcome is presented in Table 3. The bladder capacity increased significantly within the first 6 months postoperatively. The bladder capacity was recorded as volume (ml) and percent of expected capacity for age (%). According to the urinary outcome, the median (first quartile and third quartile) of the bladder capacity volume augmented from 35 (10–71) to 110 (40–160), and the percent of expected capacity for age increased from 33 (7.5–46) to 85 (36–91) within 6 months (*P* < 0.05). In addition, incontinent patients' bladder capacity was significantly smaller than that of continent or social continent patients (*p* < 0.01).

**Table 3:** Urinary function after epispadias repair.

	Bladder capacity (ml)		Expected bladder capacity (%)		Continence		
	Pre-op	After 6 months	Pre-op	After 6 months	Continence (%)	Social continence (%)	Incontinence (%)
GE	220	260	0.81	0.92	1 (100)	0 (0)	0 (0)
PE	70 (32.5–75.5)	150 (95–165)	39 (31–48)	88 (84–102)	7 (78)	2 (22)	0 (0)
PPE	7 (2.5–15)	34 (33–41.5)	5 (3–10)	26 (22–40)	0 (0)	0 (0)	4 (80%)^a^
Overall	35 (10–71)	110 (40–160)	33 (7.5–46)	85 (36–91)			
*P*-value	0.018		0.0004				

^a^ One PPE patient was waiting for continence evaluation.

The occurrence of erections in all 15 patients was confirmed by direct observation by the physician or by a parent's interview.

## Discussion

IME is a rare urogenital anomaly with an incidence of 1/117,000 (13). It remains a challenge for surgeons due to its surgical complexity and difficulty and the numerous necessary surgical procedures. The aim of epispadias restoration includes straightening of the dorsal curvature, urethral reconstruction, glanuloplasty, and penile skin closure (14). In the past few decades, a large number of surgical techniques have been introduced for BEEC repair, but few researchers have focused on the outcome of IME. Many studies have reported a combination of patients with BEEC and IME. Therefore, it is difficult to evaluate the specific results of IME as a distinct congenital urogenital anomaly (15).

Among the variants of epispadias restoration, complete disassembly of the penis is one of the most successful and popular techniques (16). In cases of BEEC, it allows movement of the corpora and urethral plate and allows for ventral placement of the neourethra and bladder neck in the pelvis. Although this technique has been used with good outcomes for the reconstruction of IME, the procedure is complex and requires skilled surgeons. Another classic surgical procedure for epispadias repair is the Cantwell–Ransley technique. The Cantwell–Ransley procedure rotates the corpus cavernosum to place the neourethra ventrally. However, compared to the complete penile disassembly procedure, the Cantwell–Ransley procedure may not increase outlet resistance and results in a low continence rate (17). The rareness of IME limits our ability to fully evaluate the outcomes of these methods. Our modification based on the complete penile disassembly procedure aims at less invasive repair and anatomical reconstruction of the existing structures, which may be sufficient. Compared to complete penile disassembly, the modification mainly keeps the distal glans attached and partially separates the corporal cavernosum, which simplifies the procedure. Compared with the Cantwell–Ransley procedure, this modification can also increase urethral resistance. Nevertheless, there are still some limitations to this modification.

Glans loss is a severe complication of epispadias repair. Hammouda reported that ischemic changes at the glans penis in the form of immediate postoperative darkening of the skin were identified in 5 of the first 10 patients (7). Husmann and Hammouda reported that darkening of the penile skin and hemiglans was identified in 12% of patients and sloughing off in 5% (7, 8). In our study, one of the first two patients experienced an ischemic change, which ultimately resulted in partial hemiglans loss. The exact frequency of ischemia is still unknown. The reason for the ischemia is iatrogenic and may be due to injury of the neurovascular bundles, which is part of the learning curve for the method. The corporeal arteries, which are different from those of the normal penis, pass through the external side of the corpora between 2 and 3 o'clock. At the glans, these bundles anastomose to each other (18). Thus, this anatomical anomaly should receive attention when degloving, rotating the corpus cavernosum for penile curvature, and freeing the urethral plate. The glans remain attached to reserve bundles anastomosed at the glans that may partially prevent sloughing off, which seems to reduce the learning curve for IME repair. Cervellione et al. (19) suggested that maintaining collateral circulation in the distal glans spongiosum may reduce the risk of penile tissue damage. In our study, partial glans atrophy may have been attributed to corporal/vascular injury during IME repair (8). Moreover, unstable hemodynamics during the newborn period may increase the risk of ischemic complications (19).

Continence is another concern of patients with IME. Of these 15 patients, 9 PE patients and 1 GE patient had continence or social continence. Four PPE patients were incontinent and needed to undergo bladder neck reconstruction. One PPE patient did not yet reach the age at which a continence evaluation can be performed. The continence rate was similar to that in a previous study (20). In contrast, other publications have reported continence levels of 0%–25% (17, 21). The high continence rate of our study may be attributed to the approach enhancing outlet resistance (17). The boundary of the urethral plate dissection and tubularization for GE and PE patients was extended up to the verumontanum, whereas for PPE patients, it was limited to the proximal urethral opening. Bladder neck reconstruction was not involved in the procedure for PPE patients, which may be a contributing factor to their incontinence. Bladder capacity is another critical factor in continence. For PE and GE patients, bladder capacity was nearly 80% of the expected value for their age, whereas PPE patients had a bladder capacity below 50% of the expected value. Achieving a bladder capacity of over 80% of the expected value may also contribute to achieving continence. Nevertheless, when discussing continence and ischemic change, IME is often considered together with BEEC. Few studies have focused on IME. A larger-scale study on IME repair is warranted to evaluate the real incidence of continence and ischemic change.

Of more concern than the frequency of ischemic changes are the reports of indispensable hypospadias, which appears to be an intrinsic complication of the complete penile disassembly procedure that can be found in 30%–70% of patients. To avoid shortening of the neourethra and ensure the vascularization of the urethral plate, El-Sherbiny and Hafez (22) suggested a modification of the procedure that involved keeping the distal portion of the urethral plate connected to the glans. However, preserving the distal attachment point of the urethral plate to the hemiglans makes glans reconstruction and transposition of the neourethra ventrally difficult (3), which increases the risk of glans dehiscence. Thus, freeing the urethral plate facilitates glans reconstruction and neoplastic urethra transposition ventrally. In addition, appropriate fixation to the apex of the glans and corpora cavernosa is helpful to avoid urethral retraction. Among the 15 patients included in this study, 2 had urethral shortening. We utilized a preputial island flap to restore the distal defect in the urethra as described in a previous study (11, 12).

Two patients had a residual dorsal curvature of approximately 10–15°. Longer follow-up is required to determine whether these patients need further repair.

There were three cases of urethral fistula postoperatively, and the patients underwent surgical repair 6 months later. In addition, no glans dehiscence or urethral stricture was found in any patient in our study. We believe that this modification simplified the complete penile disassembly procedure and that the outcome was acceptable.

## Conclusion

The outcome of our study demonstrates that this modified penile disassembly procedure is a simple, effective, and safe method for IME repair that leads to an acceptable penile appearance with acceptable complication rates. This modification not only avoids the separation of the glans but also simplifies the procedure. However, longer follow-up of additional patients is needed to evaluate the long-term effects of this modification.

## Data Availability

The original contributions presented in the study are included in the article/Supplementary Material, further inquiries can be directed to the corresponding authors.

## References

[1] Mollard P Basset T Mure PY. Male epispadias: experience with 45 cases. J Urol. (1998) 160(1):55–9. 10.1016/S0022-5347(01)63027-1 9628604

[2] Cantwell FV. I. Operative treatment of epispadias by transplantation of the urethra. Ann Surg. (1895) 22(6):689–94. 10.1097/00000658-189507000-00084 17860237 PMC1424876

[3] Mitchell ME Bägli DJ. Complete penile disassembly for epispadias repair: the Mitchell technique. J Urol. (1996) 155(1):300–4. 10.1016/S0022-5347(01)66649-7 7490875

[4] Kaefer M Andler R Bauer SB Hendren WH Diamond DA Retik AB. Urodynamic findings in children with isolated epispadias. J Urol. (1999) 162(3 Pt 2):1172–5. 10.1016/S0022-5347(01)68118-7 10458459

[5] Mouriquand PD Bubanj T Feyaerts A Jandric M Timsit M Mollard P Long-term results of bladder neck reconstruction for incontinence in children with classical bladder exstrophy or incontinent epispadias. BJU Int. (2003) 92(9):997–1001; discussion 1002. 10.1111/j.1464-410X.2003.04518.x 14632863

[6] Grady RW Mitchell ME. Complete primary repair of exstrophy. J Urol. (1999) 162(4):1415–20. 10.1016/S0022-5347(05)68327-9 10492227

[7] Hammouda HM. Results of complete penile disassembly for epispadias repair in 42 patients. J Urol. (2003) 170(5):1963–5; discussion 1965. 10.1097/01.ju.0000092227.00999.a6 14532834

[8] Husmann DA Gearhart JP. Loss of the penile glans and/or corpora following primary repair of bladder exstrophy using the complete penile disassembly technique. J Urol. (2004) 172(4 Pt 2):1696–700. discussion 1700–1. 10.1097/01.ju.0000138675.16931.cb 15371793

[9] Weber DM Schönbucher VB Landolt MA Gobet R. The pediatric penile perception score: an instrument for patient self-assessment and surgeon evaluation after hypospadias repair. J Urol. (2008) 180(3):1080–4; discussion 1084. 10.1016/j.juro.2008.05.060 18639292

[10] Bar-Yosef Y Savin Z Ekstein M Ben-David R Dekalo S Bar-Yaakov N Preoperative bladder capacity predicts social continence following bladder neck reconstruction in children born with exstrophy-epispadias complex. Eur J Pediatr Surg Off J Austrian Assoc Pediatr Surg Z Kinderchir. (2023) 33(6):510–4. 10.1055/a-2003-1823 36549335

[11] Thomalla JV Mitchell ME. Ventral preputial island flap technique for the repair of epispadias with or without exstrophy. J Urol. (1984) 132(5):985–7. 10.1016/S0022-5347(17)49978-2 6492292

[12] Hafez AT Helmy T. Complete penile disassembly for epispadias repair in postpubertal patients. Urology. (2011) 78(6):1407–10. 10.1016/j.urology.2011.06.053 21924761

[13] Dees JE. Congenital epispadias with incontinence. J Urol. (1949) 62(4):513–22. 10.1016/S0022-5347(17)68966-3 18149278

[14] Gearhart JP. Evolution of epispadias repair–timing, techniques and results. J Urol. (1998) 160(1):177–8. 10.1016/S0022-5347(01)63085-4 9628645

[15] Carrasco A Jr Vemulakonda VM. Managing adult urinary incontinence from the congenitally incompetent bladder outlet. Curr Opin Urol. (2016) 26(4):351–6. 10.1097/MOU.0000000000000296 27096718

[16] Perovic SV Vukadinovic V Djordjevic ML Djakovic NG. Penile disassembly technique for epispadias repair: variants of technique. J Urol. (1999) 162(3 Pt 2):1181–4. 10.1016/S0022-5347(01)68122-9 10458461

[17] Braga LH Lorenzo AJ Bägli DJ Khoury AE Pippi Salle JL. Outcome analysis of isolated male epispadias: single center experience with 33 cases. J Urol. (2008) 179(3):1107–12. 10.1016/j.juro.2007.10.095 18206921

[18] Acimi S Debbous L Acimi MA Khelil AL. Is there a shortening of the urethral plate in complete penile disassembly used in epispadias repair, and what is its impact on the final outcomes? J Plast Reconstr Aesthet Surg. (2018) 71(11):1637–43. 10.1016/j.bjps.2018.07.002 30154046

[19] Cervellione RM Husmann DA Bivalacqua TJ Sponseller PD Gearhart JP. Penile ischemic injury in the exstrophy/epispadias spectrum: new insights and possible mechanisms. J Pediatr Urol. (2010) 6(5):450–6. 10.1016/j.jpurol.2010.04.007 20541473

[20] Spinoit AF Claeys T Bruneel E Ploumidis A Van Laecke E Hoebeke P. Isolated male epispadias: anatomic functional restoration is the primary goal. BioMed Res Int. (2016) 2016:6983109. 10.1155/2016/6983109 27722172 PMC5046007

[21] Peters CA Gearhart JP Jeffs RD. Epispadias and incontinence: the challenge of the small bladder. J Urol. (1988) 140(5 Pt 2):1199–201. 10.1016/S0022-5347(17)42001-5 3184295

[22] El-Sherbiny MT Hafez AT. Complete repair of bladder exstrophy in boys: can hypospadias be avoided? Eur Urol. (2005) 47(5):691–4. 10.1016/j.eururo.2004.10.010 15826764

